# Clinical patterns and characteristics of midfacial fractures in western romanian population: a 10-year retrospective study

**DOI:** 10.4317/medoral.23153

**Published:** 2019-10-27

**Authors:** Paul Andrei Țenț, Raluca Iulia Juncar, Mihai Juncar

**Affiliations:** 1 Department of Oral and Maxillo-Facial Surgery, University of Oradea, Romania, Str. Piața 1 Decembrie, no.10, 410073, Romania

## Abstract

**Background:**

The aim of this study was to identify the clinical pattern of midfacial fractures and concomitant associated injuries in our geographical area, as well as to correlate them in order to determine the type of fracture with the highest incidence of associated injuries.

**Material and Methods:**

A 10-year retrospective evaluation of midfacial fractures was performed in 379 patients.

**Results:**

Zygomatic complex fractures had the highest incidence (n=242, 50%). The majority of the fractures were complete (n=561, 92.42%), closed (n=473, 84.16%) and without displacement (n=454, 80.78%) regardless of the location of the fracture line (*p*=0.014). All patients had at least one associated soft tissue injury (n=379, 100%). The most frequent associated injury was hematoma (n=308, 73.51%). Hematomas were predominant in the case of single fractures, while lacerations and excoriations were prevalent in the case of multiple fractures (*p*=0.000).

**Conclusions:**

Following trauma of the midface, patients with soft tissue hematomas will most probably have an underlying fracture with a single trajectory, while patients with lacerations will most probably have concomitant multiple bone fractures.

** Key words:**Midface, fracture, trauma, pattern, associated injuries.

## Introduction

The traumatology of the middle viscerocranium is currently a subject of the greatest importance in maxillofacial surgery, correct diagnosis and an impeccable therapeutic approach being each time a challenge for the surgeon (1). Midfacial traumas frequently present a rich and extremely varied clinical picture from one case to another, including concomitant skeletal fractures, associated soft tissue injuries, as well as eye, sinus and dentoalveolar injuries (2).

The incidence of midfacial fractures has continuously increased over the past years, tending to become in some geographical regions the most frequent emergency in specialized clinics (3).

The pattern of midfacial fractures differs depending on the trajectory and location of the fracture lines, the affected bone structures and their degree of involvement, as well as on the degree of displacement of the fractured fragments (4). The absence of current literature consensus regarding the pattern of midfacial fractures and the extremely divergent opinions of the different authors can create diagnostic confusions among specialists (5). Associated soft tissue injuries can be in certain circumstances pathognomonic for the identification of an underlying fracture line, but there are also cases when these can mask bone lesions, which frequently go unnoticed (6). The absence of an early diagnosis of midfacial fractures may have major long-term morphological, functional and aesthetic implications (6,7). Psychological disorders such as post-traumatic stress syndrome and depression frequently occur in these cases, amplifying the degree of difficulty of subsequent treatment (7). In this context, we believe that determining the pattern of midfacial fractures in our population is imperative for adopting an optimal therapeutic approach.

The aim of this retrospective study is to evaluate the clinical characteristics of midfacial fractures and their associated injuries, as well as to correlate them in order to identify the type of fracture with the highest incidence of associated injuries.

## Material and Methods

For this study, the patients admitted and treated for midfacial fractures in the Clinic of Oral and Maxillofacial Surgery I Cluj-Napoca, in the period 1 January 2002 – 31 December 2011, were available.We mention that this study was approved by Territorial Ethics Commission and have therefore been performed in accordance with ethical standards laid down in the 1964 Declaration of Helsinki and its later amendmends, reference number: 192465.

Data were extracted from the patients’ clinical observation sheets, and the following variables were monitored: the degree of bone involvement (incomplete/complete fracture), the topographic location of the fracture in the midface (Le Fort I, Le Fort II, Le Fort III, zygomatic complex, nasal bones, alveolar ridge, orbital floor, anterior wall of the maxillary sinus), the degree of displacement of the bone fragments (with displacement/without displacement), the relation of the fracture focus to the external environment (intraorally closed/open/extraorally open fracture), the type of associated injuries (contusion, excoriation, laceration, dental trauma), the presence of dental trauma (crown/root fracture, tooth avulsion/luxation).

The study inclusion criteria were: the presence of at least one fracture line in the midface, a history of an acute trauma episode, paraclinical examinations (X-ray or computed tomography) confirming the clinical diagnosis of fracture and evidencing its location and characteristics, treatment of the fracture performed in the study host institution, signing of an informed consent by all patients, through which they agreed to the use of their medical data for scientific research.

Study exclusion criteria: patient without fracture lines in the midface, fracture of a different etiology than traumatic, absence of complementary imaging investigations, treatment performed in another service, incomplete data.

Data centralization in electronic format was carried out using the Microsoft Excel software. Descriptive statistics of the evaluated cases was performed with a two decimal percentage accuracy. Statistical analysis was conducted with the MedCalc Statistical Software version 17.2 (MedCalc Software bvba, Ostend, Belgium;https://www.medcalc.org; 2017). Continuous data were expressed as mean and standard deviation, and nominal data were expressed as frequency and percentage. The frequencies of a nominal variable across the categories of another nominal variable were compared with the chi-square test. The comparison of a continuous nominal variable between two groups was performed with the T test for independent variables. A *p* value *p*<0.05 was considered statistically significant.

## Results

The study inclusion criteria were met by 379 patients of all 12645 patients treated in the host institution during the analyzed time period. These had 562 fracture lines in the midface and fulfilled the inclusion criteria in this study. Of these, 297 (78.36%) strictly had midfacial fractures, and 82 (21.64%) had both midfacial fractures and concomitant mandibular fractures. Depending on the number of fracture lines in the viscerocranium, 335 patients (88.39%) had single fractures, while 44 (11.61%) had multiple fractures.

**Figure 1:**
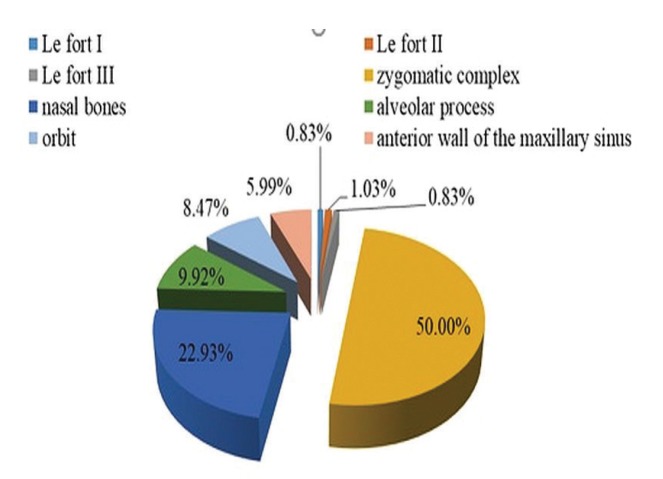
Distribution of the fracture lines depending on location.

The most frequent fractures located in the midface were zygomatic complex fractures, followed by nasal bone and maxillary alveolar ridge fractures. Le Fort I fractures had the lowest incidence. No panfacial fracture was found in this study (Fig. 1).

The majority of the fracture lines were complete n=561 (92.42%), incomplete fracture lines representing a small proportion n=46 (7.58%). Fracture fragment displacement was present only in 108 (19.22%) fracture foci, the majority being without displacement n=454 (80.78%). Closed fracture foci were predominant n=473 (84.16%), being followed by intraorally n=83 (14.77%) and extraorally open fracture foci n=6 (1.07%).

All patients had associated soft tissue injuries n=379 (100%). The most frequent associated soft tissue injury was hematoma, followed by excoriation and laceration, dentoalveolar injuries being found in a small number of cases. Among dental-periodontal injuries, the most frequent was dental avulsion n=25 (31.25%), followed by dental luxation n=24 (30.00%), crown fracture n=22 (27.50%) and root fracture n=9 (11.25%).

The incidence of associated injuries was correlated with the topographic location of the fracture trajectory in the midface (Table 1).

**Table 1:**
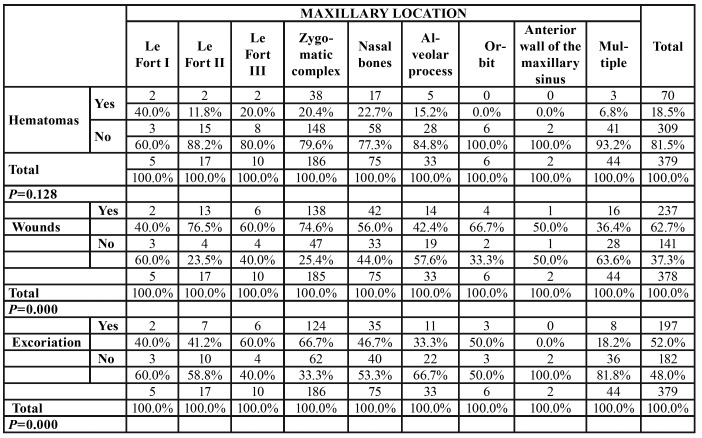
Correlation of the frequency of soft tissue injuries with the topographic location of the fracture lines.

Lacerations and excoriations had a significantly increased incidence among patients with multiple midfacial alveolar process and nasal bone fractures. This result was statistically significant (*p*=0.000). Hematomas were predominant among patients with zygomatic complex, orbital and Le Fort I, II and III fractures. However, this result was not statistically significant (*p*=0.128).

The majority of midfacial fractures had no contact with the external environment, irrespective of the fracture line trajectory (Table 2). This result was statistically significant (*p*=0.014).

**Table 2:**
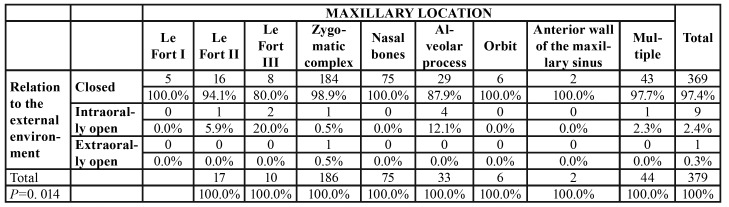
Distribution of the frequency of the relation to the external environment depending on the topographic location of the fracture lines.

The presence of hematomas was directly proportionally correlated with fracture fragment displacement, but the correlation was not statistically significant (*p*=0.469) (Table 3).

**Table 3:**
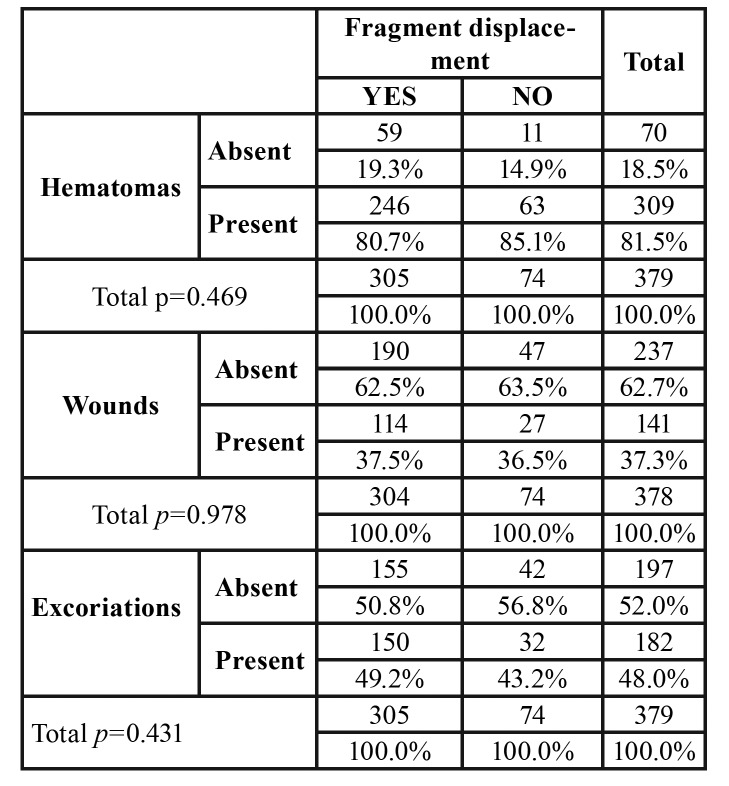
Correlation of the frequency of soft tissue injuries with the degree of fracture displacement.

## Discussion

Midfacial fractures may occur per se or in association with fractures of other cranial bone structures (6). In the current study, some of the patients had concomitant mandibular fractures, a result also reported by other authors (8-11). This can be due to the anatomical mandibular prominence in the viscerocranium, which is thus more exposed to trauma (9). The vertical mandibular ramus is also in a close anatomical relationship with the midfacial skeleton through the mandibular condyle that is part of the temporomandibular joint and the coronoid apophysis which is in a close relationship with the zygomatic bone (1,2). Thus, a wounding agent with a large contact surface that acts from lateral direction on the zygomatic complex can induce concomitant condylar, vertical mandibular ramus or coronoid apophysis fractures (2,6,9). Contrary to our results, other authors indicate a higher incidence of cranial base and cranial bone fractures in association with midfacial fractures (12,13). The mentioned differences can be explained by the fact that the pattern of craniofacial fractures depends on a multitude of factors such as the type, direction, kinetic energy of the injuring agent or the position of the head at the time of the trauma, and especially on the fracture mechanism, leading to many possible variants of association of the fracture foci (9-13).

The majority of the patients in this study had a single fracture trajectory in the midface, those with multiple fractures representing a small proportion. This result is also found in the publications of other authors (8,14,15). This can be explained by the reduced kinetic energy of the wounding agents that induced midfacial fractures in our geographical area. Previous studies conducted in our geographical area indicate the main etiological factor of maxillofacial traumas to be interpersonal violence by hitting with the fist (16). It is well known that kinetic energy developed by hitting with the fist is rarely sufficiently high to induce multiple fractures in the viscerocranium (14,15). Contrary to our results, in studies conducted in patients traumatized by wounding agents with high kinetic energy such as firearms, explosives or serious road traffic accidents, the predominance of multiple or even panfacial fractures in the midface is indicated (17-19). In this context, Romanian legislation that forbids civilian firearm possession and the fact that Romania is currently not a conflict area explain the predominance of less severe maxillofacial traumas compared to other geographical areas (16).

The most frequent midfacial fractures in this study were zygomatic complex fractures, which is also reported by other authors (3,4,6,11,12,15,20,22,23). This result can be due to the zygomatic bone prominence in the viscerocranium, which makes it susceptible to trauma (20,22,23). Also, the zygomatic complex is biomechanically the lateral weight-bearing pillar of the midface, absorbing a large part of the kinetic energy of the wounding agents (3,4). When kinetic energy exceeds the absorption capacity of the zygomatic complex, this will fracture (3,4,5). Another aspect that should not be neglected is human defense instinct. People are frequently tempted to turn their head at the moment of the trauma, avoiding in this way frontal impact in the middle of the face (11,12,15). This can explain the results obtained; however, contrary to our report, other authors indicate nasal bone fracture as having the highest incidence (1,5,16,25,26). The nasal bone prominence in sagittal plane in the facial contour explains the high incidence of fractures at this level (25,26). The exact incidence of nasal bone fractures in our geographical area is not known, because a significant part of these traumas are treated in ENT services. Also, it is known that nasal bones have a reduced biomechanical resistance to traumas, fracturing even as a result of the action of wounding agents that develop low intensity kinetic energy (5). In this study, nasal bone fractures were also found in a considerable number, representing the second most frequent topographic location. Other authors such as Ramli *et al*. (12), Runci *et al*. (27), Roccia F *et al*. (28) and Smith H *et al*. (29) indicate orbital fractures to have the highest incidence in the midface. In the current study, the fractures located in the frontal zygomatic processes and those strictly situated on the inferior orbital margin without the involvement of the orbital floor were included in the category of zygomatic complex fractures. This fact can explain the lower incidence of orbital fractures in this study compared to the results of the mentioned authors. Single location Le Fort fractures had a low incidence in the results obtained. This is in line with the majority of the specialized publications (1,4,11,20,22). It is currently unanimously accepted that Le Fort classification plays only a guiding role in clinical practice (1,4,20,22,23). Under the action of the multitude of wounding agents, the midface rarely fractures strictly along the trajectory of the fracture lines described by Le Fort; most frequently, multiple fractures and combinations of these are found distributed in various topographic locations of the facial massif (4,22,25,26). However, neither this study nor the previously mentioned publications describe the nature of the traumatic agent, its direction of action or the kinetic energy developed by it in each individual case. In this context, our explanations regarding the development of Le Fort fractures are purely speculative. Contrary to the results presented above, in studies conducted in patients from war areas, panfacial fractures are predominant (17,18).

In the current study, the majority of the fractures were complete, a result unanimously supported by the literature (2,4,6,7,12,14,15,21). The presence of nasal fossae and maxillary sinuses, as well as pneumatization of the latter with their secondary volume expansion during the course of life considerably decreases facial bone wall thickness, reducing bone cortices (2,4). Under these circumstances, an injuring agent that acts on the midface will rarely induce an incomplete fracture (21,26).

Displaced fractures in our study had a low frequency, fractures without displacement being predominant. It is known that bone fragment displacement in the midface is usually primary, occurring under the direct action of the traumatic agent and being directly proportional to its kinetic energy (6,8,14). Secondary displacement due to pterygoid and masseter muscle traction is rarely biomechanically significant in the case of the midface (14,26). Thus, it is possible that in our study the majority of the injuring agents that caused fractures at this level may not have had sufficiently high kinetic energy to induce direct bone fragment displacement. However, given the retrospective nature of the study, this fact cannot be established with certainty, the explanations being purely speculative. Contrary to our result, most authors indicate a predominance of displaced fractures in the midface (2,17,18,20,26).

The majority of the fracture foci present in our study were closed, without being contaminated from the septic oral or external environment, irrespective of the topographic location. This result was statistically significant and is also found in other publications (1,24,25,27,29). In contrast, other authors indicate a predominance of open fractures in the midface (17-20). The fact that the literature data are contradictory is not surprising, given the multitude of injuring agents that may cause midfacial fractures. The low incidence of open fractures in the current study can be best explained taking into account the social and political context of the area where the study was performed. It is known that gunshot or explosion facial traumas are particularly severe, with soft tissue laceration and exposure of underlying fractures (17,18). The opening of the fracture focus may also depend on topographic location in the midface (14). For example, zygomatic complex fractures, which are predominant in the current study, are rarely open in the absence of severe injuries with high kinetic energy, while maxillary alveolar process fractures, due to adherence of the gingival fibromucosa at this level, are intraorally open through the nature of the trauma itself (20,23).

The highest frequency among associated soft tissue injuries was that of hematoma, a result that is also found in other specialized publications (4,5,30). This is not surprising given the predominance of zygomatic complex and nasal bone fractures in this study, which are frequently accompanied by palpebral, periorbital or conjunctival hematomas (30). The predominance of hematomas in the current study evidences the reduced severity of the included traumas. Contrary to the results obtained by us, other authors indicate laceration to be the most frequent associated midfacial injury (17,18,20,22,26,29), while other authors indicate excoriation (12) or dental injuries (3,15,28). Dental injuries are found in a small number of our cases, probably due to the fact that the majority of the patients included in the study were partially or completely edentulous at the time of the trauma. These statements are only assumptions; at the time of the study, no accurate data on the dental status of each individual patient were available. Further studies on this subject are required.

Following the correlation of associated injuries with the type of fracture, the fact that excoriations and lacerations had a clearly higher incidence in the case of multiple midfacial fractures was found to be statistically significant. This is confirmed by other authors (5,17-19). Multiple fractures generally occur after high kinetic energy trauma (5,19). The kinetic energy that causes bone fractures also impacts the overlying soft tissues, the resulting injuries being more extensive (17,18). The correlation of the degree of displacement with the type of associated injuries evidenced no statistically significant differences. The development of associated midfacial injuries was probably not directly influenced by the fracture displacement, but rather depended on the kinetic energy of the injuring agent. Similar results are described by other authors (2,17,18).

The aim of this study was achieved, and the clinical characteristics of midfacial fractures as well as the type and incidence of their associated injuries can be determined in a large group of patients. Knowing the frequency of association of the type of soft tissue injury with a certain type of midfacial fracture helps to establish a rapid and complete diagnosis because in this way, the clinician will know when to suspect the presence of a fracture that can be masked by other clinical signs.

One of the most important limitations of the current study results from its retrospective nature; some of the data collected from the observation sheets could be incomplete or incorrectly recorded. In order to minimize this shortcoming, only complete observation sheets were selected, which led to the loss of a number of cases from the statistical database. Another limitation is the fact that in the midface, zygomatic complex fractures are frequently combined with orbital fractures, which makes them difficult to evaluate retrospectively. Thus, it is impossible to know precisely whether at the time of presentation, the type of fracture was correctly diagnosed and classified from a topographic point of view. This limitation can be overcome by conducting a prospective clinical study in the future.

## Conclusions

The majority of the midfacial fractures are complete, closed and without fracture fragment displacement regardless of the location of the fracture focus. The most frequent fractures are zygomatic complex fractures. The most frequent associated soft tissue injury is hematoma. The most frequent associated dental injury is dental avulsion. The presence of severe associated injuries requires a rigorous clinical and imaging examination, given the high probability of the presence of viscerocranial fractures.
